# Addressing common biases in the evaluation of lifetime alcohol consumption patterns and dementia risk: the EPIC-Spain dementia cohort

**DOI:** 10.3389/fnut.2025.1671047

**Published:** 2025-10-14

**Authors:** José M. Huerta, Sandra M. Colorado-Yohar, M. Encarnación Andreu-Reinón, Olatz Mokoroa, Mikel Tainta, Marcela Guevara, Alba Gasque, Jesús Castilla, Dafina Petrova, Marta Crous-Bou, Raúl Zamora-Ros, María José Sánchez, María Dolores Chirlaque

**Affiliations:** ^1^CIBER Epidemiología y Salud Pública (CIBERESP), Madrid, Spain; ^2^Department of Epidemiology, Murcia Regional Health Council, Murcia, Spain; ^3^Murcia Biomedical Research Institute (IMIB), Murcia, Spain; ^4^Research Group on Demography and Health, National Faculty of Public Health, University of Antioquia, Medellín, Colombia; ^5^Virgen de la Arrixaca Clinical University Hospital, Murcia Health Service, Murcia, Spain; ^6^Biogipuzkoa Health Research Institute, Donostia/San Sebastián, Spain; ^7^Sub-Directorate for Public Health and Addictions of Gipuzkoa, Ministry of Health of the Basque Government, San Sebastián, Spain; ^8^Osakidetza, Organización Sanitaria Integrada (OSI) Goierri-Urola Garaia, País Vasco, Spain; ^9^Instituto de Salud Pública y Laboral de Navarra, Pamplona, Spain; ^10^Navarra Institute for Health Research (IdiSNA), Pamplona, Spain; ^11^Escuela Andaluza de Salud Pública (EASP), Granada, Spain; ^12^Instituto de Investigación Biosanitaria ibs.GRANADA, Granada, Spain; ^13^Servicio de Oncología Médica, Hospital Universitario Virgen de las Nieves, Granada, Spain; ^14^Unit of Nutrition and Cancer, Cancer Epidemiology Research Programme, Catalan Institute of Oncology (ICO), Bellvitge Biomedical Research Institute (IDIBELL), Barcelona, Spain; ^15^Department of Health & Social Sciences, University of Murcia, Murcia, Spain

**Keywords:** alcohol consumption, dementia, Alzheimer's disease, observational study, EPIC, competing risks, sick-quitter bias, Mediterranean cohort

## Abstract

**Background:**

Alcohol consumption has been described to exhibit a J-shaped relationship with dementia risk, but previous observations may be partly biased due to “sick-quitters” and competing risks of death.

**Objective:**

To examine the association between baseline and lifetime alcohol consumption and the risk of dementia and subtypes in a large Mediterranean cohort, accounting for lifetime drinking patterns, potential confounding, and competing risks of death.

**Methods:**

Prospective study of 30,211 participants, 29–69 years at recruitment (1992–1996), from the EPIC-Spain dementia cohort. Alcohol intake was assessed using a validated dietary history and retrospective questionnaires covering ages 20, 30, and 40 years. Dementia cases (*n* = 1,114) were ascertained through linkage with healthcare and mortality databases and individual medical record review over a mean follow-up of 22.8 years. Multivariate competing risk models were used to estimate sub-hazard ratios (sHRs) for dementia by categories of baseline and lifetime alcohol consumption, using lifetime abstainers as the reference group.

**Results:**

Mean lifetime alcohol consumption was 41.9 and 4.4 g/d in men and women, respectively. No significant associations were found between baseline or lifetime alcohol consumption and risk of overall dementia (sHR_currentvs.never_ = 0.96, 95% CI: 0.82, 1.13; sHR_evervs.never_ = 0.96, 95% CI: 0.82, 1.11), Alzheimer's disease, or non-Alzheimer subtypes. These null findings remained consistent across strata of sex, BMI or smoking categories, and by beverage type. Sensitivity analyses excluding mis-reporters of energy intake or low-quality diagnoses yielded similar results.

**Conclusions:**

In this large prospective cohort with over 1,100 dementia cases and long-term follow-up, alcohol consumption was not significantly associated with dementia risk. These findings challenge the notion of a protective effect of moderate drinking and warrant continued investigation using methodologically rigorous approaches to clarify the role of alcohol dose, timing, and pattern on dementia risk.

## Introduction

Dementia is an incurable neurodegenerative syndrome of complex etiology, with both genetic and environmental determinants. Dementia and Alzheimer disease are leading causes of disability among the elderly worldwide, affecting around 57 million people (mainly in low- and middle-income countries) (1) and imposing severe economical and personal costs to patients and their families, the health systems, and society overall (2). Although incidence trends may be stabilizing or even declining in some Western regions (3–5), given the population aging dynamics the absolute number of people with dementia is projected to increase dramatically in the next decades, reaching 150 millions by 2050, unless effective prevention measures are implemented (6). Age is the most important factor to determine the development of mild cognitive impairment (MCI) and dementia. However, dementia –or Alzheimer's disease, which accounts for two-thirds of all dementia cases–, are not inevitable consequences of aging. The 2024 report of the *Lancet* Commission on dementia prevention, intervention and care estimated that up to 45% of dementia cases might be prevented by acting on 14 modifiable risk factors, including excessive alcohol consumption (2). Despite the population attributable fraction of overall dementia due to increased alcohol consumption is low, estimated as less than 1% (2, 7), the absolute number of preventable cases is not negligible due to the increasing prevalence of the disease.

The role of alcohol intake in the development of dementia is a topic of ongoing research. Alcohol has been reported to exhibit both harmful and protective effects on dementia incidence, depending on the dose and consumption pattern (8, 9). While heavy alcohol consumption is associated with higher risk of cognitive decline and dementia (2, 10–12), some observational studies have suggested that low-to-moderate alcohol intake may be protective, not only against dementia (13–15) but also for Parkinson's disease (16), coronary heart disease (17, 18) or premature mortality (19–21). Most previous reviews of observational studies, including dose-response meta-analyses of prospective studies, have suggested a U- or J-shaped curve for the association between alcohol consumption and dementia incidence (8, 10, 15, 22), with light to moderate drinkers (generally, 1–21 drinks/week for men and 1–14 drinks/week for women) showing around 30%−40% lower risk of dementia (14, 15). However, the evidence is yet insufficient since randomized controlled trials evaluating mid- to late-life alcohol consumption in relation to dementia risk are impractical due to ethical restrictions, whereas observational studies are prone to confounding and bias, and should be carefully designed (23, 24). Sick-quitters (people quitting alcohol consumption because of older age or underlying health problems) are at higher risk of chronic disease and premature mortality than lifetime abstainers (23, 25, 26), and grouping them together in a mixed category could artefactually distort effect estimates, causing the group of non-drinkers to be in a worse health condition overall (27, 28). However, only a minority of previous systematic reviews accounted for this potential bias by distinguishing former from never drinkers (10, 13). On the other hand, it is likely that other health risks would “compete” with dementia incidence when assessing the effects of alcohol consumption, so that drinkers may be less likely to develop dementia or Alzheimer's disease because a larger proportion of them would die prematurely of other chronic conditions.

In a previous study within the EPIC-Spain dementia cohort, alcohol consumption was inversely associated with dementia incidence in a univariate analysis (29). The aim of the present study was to assess the marginal association of alcohol consumption and dementia risk in multivariate models adjusting for potential confounders, addressing biases common to observational studies such as the “sick quitter” bias, and in the presence of competing risks due to non-dementia fatal events. The prospective nature of the analysis, lifetime assessment of alcohol intake, and long follow-up would allow for less-biased estimates of the relationship between alcohol consumption (amount, pattern, and beverage type) and dementia incidence in a middle-age Mediterranean population.

## Methods

### Study sample

The European Prospective Investigation into Cancer and Nutrition (EPIC) project is an ongoing multicenter epidemiological study involving over half a million participants across 10 European countries (30). The EPIC-Spain dementia cohort was established from four out of the five centers integrating the EPIC-Spain cohort (namely, Gipuzkoa, Navarra, Murcia, and Granada). The resulting study sample comprised 32,895 men and women, aged 29 to 69 years at enrollment (mean age 49 ± 8 years), who were recruited between 1992 and 1996, primarily consisting of blood donors (=60%), civil servants, and the general population (31). Exclusion criteria encompassed pregnancy, breastfeeding, and physical or mental incapacity. Detailed information on diet, lifestyles, clinical and reproductive history, and anthropometry was collected for each participant at recruitment. For the present analysis, participants with prevalent dementia (*n* = 1), missing data on alcohol consumption (*n* = 252), or prevalent diabetes, cardiovascular disease or cancer (*n* = 2,431) were further excluded, leaving a final study sample of *N* = 30,211 individuals.

The EPIC study protocol was approved by the IARC (International Agency for Research on Cancer) Ethics Committee. All participants voluntarily agreed to participate and gave written informed consent. The current research has been conducted following the principles of the World Medical Association Declaration of Helsinki and the paper was written in accordance to the *Strengthening the Reporting of Observational Studies in Epidemiology (STROBE)* statement (https://www.equator-network.org/reporting-guidelines/strobe).

### Assessment of diet and alcohol consumption

A validated diet history (DH) method was used to assess habitual dietary intake over the past 12 months (32). Participants were interviewed face-to-face by trained dietitians to report the type, frequency, cooking method, and portion size of all foods consumed during a typical week of the previous year, taking seasonal and daily variation (work days/weekend) into account. Total energy, macro- and micronutrient intakes were calculated according to the EPIC Nutrient DataBase (ENDB), which harmonized country-specific food composition tables compiled across participating countries (33).

Baseline alcohol consumption was obtained from the DH questionnaire, distinguishing the type of beverage consumed: wine (red, white, rosé), fortified wine, beer, cider, spirits, brandy, aniseed drinks, liqueurs, and cocktails. The daily amount of alcohol consumed, in grams/day, was computed as the sum of the products of the reported amount per drink (in ml) x number of drinks/day x ethanol content (%) for each type of beverage consumed. Further information on past alcohol consumption was collected at recruitment using a specific questionnaire on habitual consumption of wine, beer or cider and liquor at the age of 20, 30, and 40 years (when applicable). Lifelong average alcohol consumption was then computed (in g/day) as a weighted average of alcohol consumption at different decades of life, taking into account both the amount consumed and the sum of time (in years) during which participants had been consuming alcohol. Information on past alcohol consumption also provided guidance in distinguishing teetotalers from former drinkers.

Average baseline and lifetime alcohol consumption variables in g/d were then used to categorize participants using sex-specific cut-offs, as: “never drinkers,” “former drinkers,” “(0, 6] g ethanol/d in men/(0, 3] g/d in women,” “(6, 12] g ethanol/d in men/(3, 12] g/d in women,” “(12, 24] g/d,” “(24, 60] g/d,” “(60, 96] g/d in men/>60 g/d in women,” or “>96 g/d in men,” as detailed elsewhere (34). Finally, a categorical variable was created to reflect the lifetime pattern of alcohol intake with the following 7 levels: “never drinkers,” “former light (never-heavy) drinkers,” “former heavy drinkers,” “light drinkers” (always (0, 6] g/d), “never heavy drinkers,” “periodically heavy drinkers,” and “always heavy drinkers,” with heavy drinking defined as ≥30 g/d of alcohol in women or ≥60 g/d in men (34).

### Assessement of other covariates

Questionnarie data on socio-demographic, lifestyle, clinical, and reproductive variables were collected during personal interviews, and a physical examination was carried out to obtain anthropometric information (height, weight, waist, and hip circumferences) according to standard procedures. Data was gathered on sex, age, highest educational level and smoking habit, whereas the EPIC-PAQ questionnaire was used to assess weekly hours spent in recreational and hoseuhold physical activities (35). History of chronic disease, such as cancer, cardiovascular disease, diabetes, hypertension or hyperlipidemia was self-reported, and women also declared ever use of oral contraceptives or hormonal replacement therapy. Body mass index was estimated as weight (in kg) divided by squared height (in m). Finally, the adapted relative Mediterranean Diet score (arMED) was computed as defined by Buckland et al. (36). The arMED score is a modification of the original rMED score, excluding the alcohol component.

### Case ascertaiment and validation

Incident dementia cases occurring in the cohort were ascertained following a two-step protocol, as detailed elsewhere (37), until end of follow-up, depending on the center: November 30, 2016 for Murcia, December 31, 2017 for Gipuzkoa and Navarra, and June 30, 2021 for Granada. Briefly, potential cases of dementia were identified by record-linkage with healthcare and mortality databases using dementia-related codes from the *International Classification of Diseases and Related Health Problems (ICD)*, 9th (codes: 290, 331) and 10th (codes: F00-F03, G30) editions, the *International Classification of Primary Care (ICPC)*, 2nd edition (codes: P20, P70, N29, N99) and the *Anatomical Therapeutic Chemical classification system (ATC)* (codes: N06DA02, N06DA03, N06DDA04, N06DX01). A panel of neurologists then revised the available medical records (in electronic or paper forms) of all potential cases identified, and established the diagnosis of dementia and, when possible, the sub-type according to the reliability of information from the sources available (primary care, outpatient and hospital medical records, drug prescriptions, diagnostic tests, and mortality databases). The incidence of the disease was deemed to be based on “high-quality” information when a diagnosis of dementia appeared in a neurological report. When a dementia diagnosis was found on a report from another medical specialist (such as a psychiatrist, a geriatric specialist or a physician) it was considered to rely on “medium-quality” information. Finally, data was considered as “low-quality” when cases were ascertained based solely on diagnostic codes. The validation study showed that diagnosis based on a combination of codes, including P70, ICD codes and/or anti-dementia drugs had very high sensitivity and specificity in the identification of dementia cases (93.1 and 96.8%, respectively) when compared with the expert revision of medical histories of the participants as the gold standard (37). The type of dementia was classified as Alzheimer (based on sufficient clinical information, including mixed dementia –Alzheimer's disease with cerebrovascular disease) and non-Alzheimer (all other types).

### Statistical analyses

Descriptive statistics of the study cohort, including sociodemographic, anthropometric, lifestyle, and clinical variables were estimated by categories of lifetime alcohol consumption as means and standard deviations or absolute and relative frequencies for continuous and categorical variables, as appropriate. Risks of dementia were estimated using Fine & Gray models with age as the timescale and non-dementia deaths as competing events. Person-time was computed for each individual from recruitment until date of dementia diagnosis, loss to follow-up, death or study end, whichever occurred first. Sub-hazard ratios and 95% confidence intervals (95%CI) of dementia or dementia sub-type were estimated for categories of baseline or lifetime alcohol intake defining the group of “never drinkers” as the reference category in all analyses to limit the potential “sick-quitter” bias. For the same reason, continuous analyses of average baseline or lifetime alcohol consumption were conducted excluding former drinkers. When modeled as continuous, alcohol intake variables were transformed using restricted cubic splines (RCS) with 3 degrees of freedom and the reference was set at 0 g/d of intake. Model 1 was adjusted by center, sex, and educational level. Model 2 also included energy intake, excluding energy from alcohol (continuous, transformed using RCS), smoking (“never,” “former,” “current”), BMI (categorical, “ < 25,” “25–29.99,” “≥30” kg/m^2^), BMI x sex interaction, waist circumference (binary, ≥102/88 vs. < 102/88 cm in men/women), household (MET·h/week) and recreational physical activity (MET·h/week), history of hypertension or hyperlipidemia, and the arMED score (continuous). Lifetime models were further adjusted by duration of alcohol consumption and time since quitting alcohol (years). Missing values in covariates (< 1%) were either imputed (continuous) or assigned an indicator category (categorical).

Stratified models by sex, smoking, and excess body weight (BMI≥25 kg/m^2^) were run to assess potential effect modification by these factors. Sensitivity analyses were conducted: (a) by excluding mis-reporters of energy intake (pTEE method) (38); (b) by restricting the analysis to participants in their mid-life (age between 45 and 65 years); and (c) by considering only cases with a high confidence in their diagnosis (i.e., based on complete neurological information) (37). Reverse causation was unlikely, as over 99% of dementia cases occurred after 5 years of follow-up.

## Results

After a mean of 22.8 ± 4.0 years of follow-up, a total of 690,345 person-years and 1,114 incident dementia cases were ascertained (3.7% of the cohort, 66.7% among women). Mean lifetime alcohol consumption was 41.9 g/d in men and 4.4 g/d in women, whereas baseline alcohol consumption amounted to 28.6 and 4.2 g/d in men and women, respectively. Table 1 shows main baseline characteristics of lifetime alcohol consumption groups. As shown, the abstainers group was comprised by a large majority of women (94%) and never smokers (77%), and together with former drinkers, they exhibited higher mean household physical activity, and Mediterranean diet score, and included a larger proportion of post-menopausal women. On the other hand, lifetime alcohol consumption groups correlated positively with educational level, waist circumference, current smoking, recreational physical activity, total energy intake, and oral contraceptive use (among women). A similar pattern was observed when ranking participants according to their average alcohol intake at baseline (Supplementary Table 1).

**Table 1:** Baseline characteristics of participants from the EPIC-Spain dementia cohort (*N* = 30,211) by lifetime alcohol consumption categories.

**Variables**	**Lifetime alcohol consumption categories (g/d)**													
	**Never drinkers** ***N*** = **6,386**		**Former drinkers** ***N*** = **4,030**		**(0, 6] (m)/(0, 3] (w)** ***N*** = **4,460**		**(6, 12] (m)/(3, 12] (w)** ***N*** = **4,601**		**(12–24]** ***N*** = **3,496**		**(24–60]** ***N*** = **4,561**		**(**>**60)** ***N*** = **2,677**	
Lifetime alcohol consumption (g/day), mean (s.d.)	0		8.8	(18.7)	1.3	(1.3)	7.1	(2.5)	17.2	(3.3)	39.5	(10.3)	91.7	(32.5)
Baseline alcohol consumption (g/day), mean (s.d.)	0		0		2.3	(3.1)	7.7	(6.2)	16.4	(10.0)	31.7	(18.2)	58.8	(34.0)
Age at recruitment (y), mean (s.d.)	49.0	(8.4)	49.6	(8.1)	48.0	(8.1)	47.5	(8.0)	48.8	(7.8)	50.1	(7.2)	51.2	(6.8)
Women, *n* (%)	5,989	(93.8)	2,997	(74.4)	3,702	(83.0)	3,891	(84.6)	1,889	(54.0)	369	(8.1)	5	(0.2)
Secondary education or higher, *n* (%)	1,134	(17.8)	880	(21.8)	1,143	(25.6)	1,304	(28.3)	1,122	(32.1)	1,579	(34.6)	695	(26.0)
Body mass index (kg/m^2^), mean (s.d.)	28.8	(4.9)	28.4	(4.5)	28.0	(4.5)	27.3	(4.2)	27.5	(3.9)	28.2	(3.3)	28.9	(3.4)
Waist circumference (cm), mean (s.d.)	88.9	(11.9)	91.0	(11.9)	89.3	(11.8)	88.0	(11.4)	92.1	(11.8)	98.4	(9.6)	101.2	(9.0)
**Cigarette smoking**, ***n*** **(%)**														
Never smoker	4,898	(76.7)	2,482	(61.6)	3,028	(67.9)	2,788	(60.6)	1,674	(47.9)	1,376	(30.2)	596	(22.3)
Former smoker	544	(8.5)	669	(16.6)	565	(12.7)	690	(15.0)	746	(21.3)	1,293	(28.4)	641	(23.9)
Current smoker	942	(14.8)	878	(21.8)	863	(19.4)	1,120	(24.3)	1,075	(30.8)	1,890	(41.4)	1,440	(53.8)
Recreational physical activity (MET·h/week), mean (sd)	24.7	(20.3)	26.8	(22.2)	25.0	(21.7)	26.3	(21.4)	28.5	(24.3)	30.8	(26.3)	29.9	(25.6)
Household physical activity (MET·h/week), mean (sd)	101.9	(47.4)	80.8	(53.1)	88.4	(51.4)	84.6	(50.8)	59.8	(52.9)	23.4	(30.7)	16.7	(21.2)
Total energy intake (kcal/day), mean (s.d.)	1,774	(565)	2,007	(636)	1,963	(591)	2,089	(615)	2,322	(627)	2,649	(663)	2,999	(735)
Energy from protein (%), mean (s.d.)	19.7	(3.5)	20.0	(3.3)	19.3	(2.9)	19.2	(2.7)	19.0	(2.6)	18.7	(2.4)	18.1	(2.5)
Energy from carbohydrates (%), mean (s.d.)	44.3	(6.5)	43.7	(6.6)	42.6	(6.0)	41.2	(6.0)	39.7	(6.1)	37.9	(6.3)	34.6	(6.3)
Energy from lipids (%), mean (s.d.)	36.0	(6.1)	36.3	(6.1)	37.2	(5.6)	36.8	(5.6)	36.0	(5.6)	34.8	(5.5)	33.6	(5.7)
Mediterranean diet score, mean (s.d.)^1^	8.3	(2.7)	8.3	(2.6)	8.2	(2.6)	8.1	(2.6)	8.0	(2.6)	8.1	(2.5)	7.7	(2.3)
Hypertension, *n* (%)	1,298	(20.3)	935	(23.2)	800	(17.9)	649	(14.1)	607	(17.4)	863	(18.9)	631	(23.6)
Hyperlipidemia, *n* (%)	949	(14.9)	784	(19.5)	618	(13.9)	702	(15.3)	681	(19.5)	1,087	(23.8)	776	(29.0)
Post-menopausal, *n* (%)^2^	2,409	(40.2)	1,218	(40.6)	1,255	(33.9)	1,214	(31.2)	639	(33.8)	94	(25.5)	1	(20.0)
Oral contraceptive use (ever), *n* (%)^2^	2,305	(38.5)	1,197	(39.9)	1,663	(44.9)	1,825	(46.9)	855	(45.3)	182	(49.3)	3	(60.0)
Hormonal replacement therapy use (ever), *n* (%)^2^	550	(9.2)	294	(9.8)	306	(8.3)	361	(9.3)	171	(9.1)	33	(8.9)	0	–

^1^Adapted relative Mediterranean diet score (arMED).

^2^Women only.

**Figure 1:**
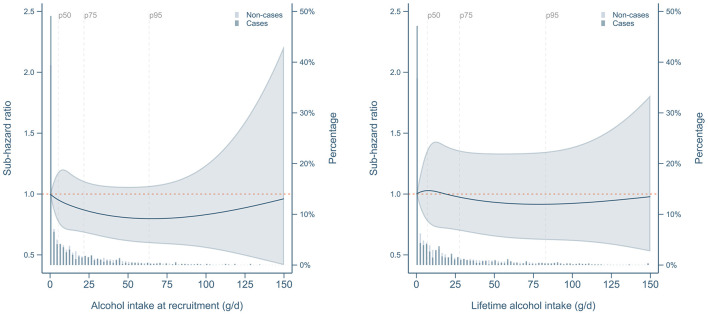
Risk of overall dementia according to baseline and mean lifetime alcohol consumption. Dementia risks were estimated by means of sub-hazard ratios derived from Fine & Gray competing risk models with age as the timescale and non-dementia deaths as competing events. Alcohol intake variables were transformed using restricted cubic splines with 3 degrees of freedom and equally spaced knots. Models were adjusted by center, sex, educational level, energy from non-alcoholic sources, body mass index category (normal weight, overweight, obese), sex x body mass index interaction, elevated waist circumference (>102 cm (men)/>88 cm (women)), household and recreational physical activity (METh/week), self-reported hypertension or hyperlipidemia, and adapted relative Mediterranean Diet score (arMED). Lifetime models were further adjusted by duration of alcohol consumption and time since quitting alcohol (years). Reference was set at 0 g/d of alcohol consumption. Former drinkers were excluded.

Main results for the association of alcohol consumption with dementia risk pointed to null associations for lifetime or baseline intake, or lifetime drinking pattern (Table 2). Sub-hazard ratios from competing-risk models were not significantly different for any considered group (including current vs. never or ever vs. never drinkers) as compared to the reference category of lifelong abstainers either in crude or adjusted models. Results remained null in separate analyses by sex (Table 3) or dementia sub-type (Table 4). Of note, most point estimates were below the unit, yet not statistically significant, as also shown in Figure 1, Supplementary Figures S1, S4. A lower risk of overall dementia was suggested for lifetime alcohol consumption among ever smokers and for alcohol intake at recruitment in the normal weight group, but those inverse associations only arose when conducting continuous analyses based on RCS (Supplementary Figures S2, S3) and were not consistent when using a categorical approach (Supplementary Tables 3, 4). Furthermore, there was no evidence that the risk of dementia could vary according to the type of alcohol consumed (wine, beer or liquor) as shown in Supplementary Figure S5.

**Table 2:** Risk of overall dementia according to lifetime and baseline alcohol consumption categories in the EPIC-Spain dementia cohort.

**Alcohol consumption**	**Person-years**	**Cases**	**Model 1**		**Model 2**	
			**SHR**	**95% CI**	**SHR**	**95% CI**
**Average baseline alcohol consumption (g/d)**						
Never drinkers	150,741	312	1 (ref.)		1 (ref.)	
Former drinkers	91,769	164	0.93	(0.77, 1.13)	0.95	(0.78, 1.16)
(0, 6] (m)/(0, 3] (w)	129,147	202	0.94	(0.78, 1.14)	0.96	(0.80, 1.16)
(6, 12] (m)/(3, 12] (w)	101,054	148	0.99	(0.81, 1.22)	1.03	(0.83, 1.26)
(12, 24]	84,593	107	0.85	(0.67, 1.08)	0.88	(0.70, 1.12)
(24, 60]	100,939	134	0.85	(0.66, 1.08)	0.87	(0.68, 1.12)
(60, 96] (m)/>60 (w)	24,843	34	0.81	(0.55, 1.19)	0.82	(0.55, 1.22)
>96 (m)	7,260	13	0.97	(0.55, 1.72)	0.97	(0.54, 1.75)
Current vs. never	598,576	950	0.94	(0.80, 1.10)	0.96	(0.82, 1.13)
**Average lifetime alcohol consumption (g/d)**						
Never drinkers	150,741	312	1 (ref.)		1 (ref.)	
Former drinkers	91,769	164	0.93	(0.77, 1.14)	0.95	(0.78, 1.16)
(0, 6] (m)/(0, 3] (w)	104,107	158	0.93	(0.77, 1.13)	0.95	(0.78, 1.15)
(6, 12] (m)/(3, 12] (w)	105,322	132	0.89	(0.72, 1.10)	0.92	(0.74, 1.14)
(12, 24]	78,868	128	1.09	(0.87, 1.36)	1.13	(0.90, 1.43)
(24, 60]	101,689	129	0.82	(0.63, 1.07)	0.85	(0.65, 1.11)
(60, 96] (m)/>60 (w)	39,726	60	0.81	(0.58, 1.14)	0.83	(0.59, 1.17)
>96 (m)	18,124	31	0.93	(0.61, 1.40)	0.95	(0.62, 1.45)
Ever vs. never	690,345	1,114	0.93	(0.81, 1.08)	0.96	(0.82, 1.11)
**Lifetime pattern of alcohol consumption**						
Never drinkers	150,741	312	1 (ref.)		1 (ref.)	
Former light drinkers	84,518	152	0.94	(0.77, 1.15)	0.96	(0.78, 1.17)
Former heavy drinkers	7,251	12	0.80	(0.44, 1.45)	0.83	(0.45, 1.50)
Light drinkers	69,410	119	1.01	(0.82, 1.25)	1.02	(0.83, 1.26)
Never heavy drinkers	265,273	356	0.90	(0.76, 1.07)	0.93	(0.78, 1.11)
Periodically heavy drinkers	93,246	138	0.87	(0.68, 1.12)	0.90	(0.70, 1.16)
Always heavy drinkers	19,907	25	0.75	(0.48, 1.16)	0.75	(0.48, 1.19)

SHR, Sub-hazard ratio; CI, confidence Interval.

Model 1: adjusted by center, sex, and educational level.

Model 2: model 1 plus adjustment by energy intake (alcohol excluded), smoking, BMI categories, sex x BMI interaction, elevated waist circumference, household and recreational physical activity, hypertension, hyperlipidemia, and Mediterranean diet score (arMED).

**Table 3:** Risk of overall dementia according to lifetime and baseline alcohol consumption categories in the EPIC-Spain dementia cohort, by sex.

**Alcohol consumption**	**Men**				**Women**			
	**Person-years**	**Cases**	**SHR**	**95% CI**	**Person-years**	**Cases**	**SHR**	**95% CI**
**Average baseline alcohol consumption (g/d)**								
Never drinkers	9,039	14	1 (ref.)		141,702	298	1 (ref.)	
Former drinkers	22,552	39	1.02	(0.55, 1.89)	69,217	125	0.95	(0.77, 1.18)
(0, 6] (m)/(0, 3] (w)	34,523	44	0.89	(0.48, 1.65)	94,624	158	1.01	(0.83, 1.23)
(6, 12] (m)/(3, 12] (w)	25,239	42	1.20	(0.64, 2.23)	75,815	106	1.00	(0.79, 1.26)
(12, 24]	44,378	66	1.08	(0.60, 1.94)	40,214	41	0.77	(0.55, 1.08)
(24, 60]	85,107	119	0.99	(0.55, 1.76)	15,832	15	0.76	(0.45, 1.30)
(60, 96] (m)/>60 (w)	24,272	34	0.95	(0.49, 1.83)	571	0	–	
>96 (m)	7,260	13	1.15	(0.52, 2.54)	–			
Current vs. never	229,818	332	1.05	(0.60, 1.84)	368,758	618	0.97	(0.81, 1.15)
**Average lifetime alcohol consumption (g/d)**								
Never drinkers	9,039	14	1 (ref.)		141,702	298	1 (ref.)	
Former drinkers	22,552	39	1.01	(0.54, 1.88)	69,217	125	0.96	(0.77, 1.19)
(0, 6] (m)/(0, 3] (w)	17,511	21	0.85	(0.43, 1.67)	86,596	137	0.97	(0.79, 1.19)
(6, 12] (m)/(3, 12] (w)	16,284	23	1.16	(0.59, 2.25)	89,038	109	0.90	(0.71, 1.14)
(12, 24]	35,984	62	1.34	(0.73, 2.43)	42,883	66	1.06	(0.80, 1.41)
(24, 60]	93,266	121	0.92	(0.52, 1.63)	8,423	8	0.86	(0.41, 1.79)
(60, 96] (m)/>60 (w)	39,610	60	0.92	(0.50, 1.71)	116	0	–	
>96 (m)	18,124	31	1.06	(0.54, 2.08)	–			
Ever vs. never	252,370	371	1.02	(0.58, 1.77)	437,975	743	0.96	(0.82, 1.12)
**Lifetime pattern of alcohol consumption**								
Never drinkers	9,039	14	1 (ref.)		141,702	298	1 (ref.)	
Former light drinkers	16,706	29	1.05	(0.55, 1.99)	67,812	123	0.95	(0.77, 1.18)
Former heavy drinkers	5,845	10	0.93	(0.40, 2.15)	1,405	2	0.78	(0.20, 3.08)
Light drinkers	9,169	12	0.91	(0.42, 1.97)	60,241	107	1.04	(0.83, 1.29)
Never heavy drinkers	112,902	159	1.04	(0.59, 1.84)	152,371	197	0.92	(0.75, 1.12)
Periodically heavy drinkers	80,198	122	1.00	(0.56, 1.79)	13,048	16	0.91	(0.54, 1.53)
Always heavy drinkers	18,509	25	0.89	(0.44, 1.80)	1,397	0	–	

SHR, Sub-hazard ratio; CI, confidence Interval.

Models adjusted by center, educational level, energy intake (alcohol excluded), smoking, BMI categories, elevated waist circumference, household and recreational physical activity, hypertension, hyperlipidemia, and Mediterranean diet score (arMED).

**Table 4:** Risk of dementia according to lifetime and baseline alcohol consumption categories in the EPIC-Spain dementia cohort, by type of dementia.

**Alcohol consumption**	**Person-years**	**Alzheimer**			**Non-Alzheimer**		
		**Cases**	**SHR**	**95% CI**	**Cases**	**SHR**	**95% CI**
**Average baseline alcohol consumption (g/d)**							
Never drinkers	150,741	232	1 (ref.)		80	1 (ref.)	
Former drinkers	91,769	115	0.95	(0.75, 1.20)	49	0.97	(0.67, 1.40)
(0, 6] (m)/(0, 3] (w)	129,147	141	0.94	(0.75, 1.17)	61	1.02	(0.72, 1.46)
(6, 12] (m)/(3, 12] (w)	101,054	103	1.02	(0.80, 1.31)	45	1.05	(0.71, 1.54)
(12, 24]	84,593	73	0.88	(0.66, 1.17)	34	0.92	(0.60, 1.39)
(24, 60]	100,939	81	0.78	(0.57, 1.07)	53	1.07	(0.70, 1.66)
(60, 96] (m)/>60 (w)	24,843	21	0.75	(0.46, 1.23)	13	1.00	(0.52, 1.93)
>96 (m)	7,260	8	0.89	(0.42, 1.87)	5	1.17	(0.44, 3.10)
Current vs. never	598,576	659	0.94	(0.78, 1.14)	291	1.03	(0.76, 1.39)
**Average lifetime alcohol consumption (g/d)**							
Never drinkers	150,741	232	1 (ref.)		80	1 (ref.)	
Former drinkers	91,769	115	0.94	(0.75, 1.20)	49	0.99	(0.68, 1.43)
(0, 6] (m)/(0, 3] (w)	104,107	113	0.93	(0.74, 1.17)	45	0.98	(0.68, 1.43)
(6, 12] (m)/(3, 12] (w)	105,322	93	0.92	(0.71, 1.18)	39	0.94	(0.63, 1.41)
(12, 24]	78,868	88	1.13	(0.86, 1.48)	40	1.16	(0.76, 1.78)
(24, 60]	101,689	75	0.72	(0.51, 1.00)	54	1.17	(0.73, 1.87)
(60, 96] (m)/>60 (w)	39,726	39	0.77	(0.51, 1.16)	21	1.02	(0.56, 1.88)
>96 (m)	18,124	19	0.82	(0.48, 1.39)	12	1.28	(0.62, 2.66)
Ever vs. never	690,345	774	0.95	(0.79, 1.13)	340	1.00	(0.75, 1.33)
**Lifetime pattern of alcohol consumption**							
Never drinkers	150,741	232	1 (ref.)		80	1 (ref.)	
Former light drinkers	84,518	110	0.97	(0.77, 1.23)	42	0.92	(0.63, 1.35)
Former heavy drinkers	7,251	5	0.51	(0.21, 1.27)	7	1.52	(0.67, 3.44)
Light drinkers	69,410	87	1.02	(0.80, 1.31)	32	1.02	(0.67, 1.55)
Never heavy drinkers	265,273	239	0.90	(0.73, 1.11)	117	1.02	(0.74, 1.40)
Periodically heavy drinkers	93,246	87	0.81	(0.59, 1.10)	51	1.14	(0.73, 1.78)
Always heavy drinkers	19,907	14	0.60	(0.33, 1.08)	11	1.17	(0.57, 2.39)

SHR, Sub-hazard ratio; CI, confidence Interval.

Models adjusted by center, sex, educational level, energy intake (alcohol excluded), smoking, BMI categories, sex x BMI interaction, elevated waist circumference, household and recreational physical activity, hypertension, hyperlipidemia, and Mediterranean diet score (arMED).

We conducted a series of sensitivity analyses, as follows: (i) we excluded mis-reporters of energy intake (*n* = 9,190, 30%), who might have under-reported their alcohol consumption; (ii) we restricted the analyses to participants in their middle-age at baseline, i.e., 45 to 65 years (*n* = 10,937 excluded, 36%), to focus on the association of mid-life alcohol intake habit and dementia risk; and (iii) we excluded dementia cases identified on the basis of insufficient clinical data or solely on ICD, ICPC, or ATC codes (*n* = 203 cases, 18% of cases), thus restricting the analysis to cases ascertained with a high degree of confidence. While estimates were modestly attenuated in the scenarios (ii) and (iii), results remained null for all associations tested. An additional model imposing all three restrictions at once conducted on *N* = 13,373 participants yielded virtually the same results as the full-cohort model (Supplementary Table 5).

## Discussion

In this analysis from the EPIC-Spain dementia cohort, a large prospective study of midlife adults followed for over 20 years and accruing over 1,100 dementia cases, we found no overall association between baseline or lifetime alcohol intake (amount or pattern) and dementia risk. Separate analyses also showed no evidence that alcohol intake was associated with dementia subtypes (Alzheimer or non-Alzheimer), sex, or type of alcoholic beverage consumed. Alcohol variables were modeled as linear, non-linear or categorical exposures in multivariable analyses accounting for competing risks of death. Our findings do not support a protective effect of moderate or light alcohol intake against dementia in midlife free-living population, nor do they indicate a significantly elevated risk for high levels of consumption, although the prevalence of heavy drinking in our sample was low.

Dose-response meta-analyses of prospective studies have suggested a U- or J-shaped association between alcohol consumption and the risk of dementia (8, 10, 15, 22). However, most previous observational studies did not account for potential biases which could distort the study of alcohol-related outcomes, such as separating former drinkers from lifetime abstainers or considering competing risks of premature death due to high alcohol consumption (8, 14), and the large majority of them have been conducted in population over 65 years-old (15, 22). A large previous study conducted in over 40,000 middle-age participants from the Norwegian HUNT cohort was able to define a group of abstainers to address the “sick-quitter” bias, but still analyses did not account for competing causes of death (39).

Alcohol consumption is inherently a variable behavior and may change over time within individuals. Such intra-individual variability represents a challenge for single-measurement analyses, potentially obscuring alcohol-disease associations and biasing risk estimates. To try to overcome this limitation, we have incorporated a retrospective lifetime approach, evaluating both baseline and average lifetime alcohol intake, and categorizing the lifetime pattern based on reported consumption at different ages. A major strength of this approach is the ability to identify a group of lifetime abstainers, allowing an unbiased reference category and reducing the potential impact of the “sick-quitter” bias. The hypothesis that individuals reduce or cease alcohol consumption with aging, illness, or frailty was first highlighted by Shaper et al. (25) in 1988 and further explored by Fillmore et al. (27) in light of observational findings suggesting a protective effect of moderate alcohol consumption on mortality. Yet, although alcohol researchers have been aware for decades of the bias introduced by mis-classifying former drinkers in the non-drinker group, not many prospective studies have been designed to distinguish lifetime abstainers from sick-quitters—or former drinkers in general–, when studying chronic disease or premature mortality (10, 40). In an insightful systematic review, Nefsey and Collins (13) reassessed the association of alcohol consumption and dementia risk based on studies excluding alcohol quitters, and still found a significant 21% lower risk among drinkers, similar to the estimated 26% reduced risk of dementia found by Ilomaki et al. (14) when comparing light-to-moderate drinkers with non-drinkers. Langballe et al. (39) however, found that the lower risk of dementia associated with infrequent drinking (1–4 times in a fortnight) in the HUNT prospective cohort was no longer significant after adjusting for potential confounders.

Another major limitation in most of the literature on alcohol intake and dementia is the failure to account for competing risks of death. Dementia is a late event, and studying causal associations of exposure variables in this context is particularly challenging because of competing causes of death (41). Some participants may die before they ever develop dementia due to unrelated causes, which then act as competing events precluding a potential dementia outcome to be observed at all. When premature mortality is associated with the exposure under study, effect estimates could be significantly biased. As alcohol, particularly excessive alcohol consumption, is an established risk factor for mortality (21), alcohol drinkers might die prematurely of other causes before they reach the usual age of onset of dementia, and thus, it might be less likely to observe dementia-related outcomes among drinkers. We therefore used Fine and Gray competing risks models defining non-dementia deaths as competing events in order to account for this potential bias in our study, where the ascertainment of death cases was comprehensive and relied on official sources from the Spanish National Statistics Institute (INE, www.ine.es). This is the largest prospective study to account for sick-quitters and competing risks biases in the same analysis of lifetime alcohol consumption and risk of dementia. This is noteworthy, as previous results from the EPIC-Spain dementia cohort not accounting for these potential biases suggested a lower risk of overall dementia among alcohol consumers as compared to non-consumers (29). This adds to other strengths, including a lifetime evaluation of alcohol consumption, a mostly middle-age cohort, a large sample size, a long follow-up and a large set of sociodemographic, lifestyle, and anthropometric covariates to evaluate as potential confounders.

The study does not come without limitations, however. Due to its observational nature it is not free of residual or unmeasured confounding. Besides, under-reporting of alcohol consumption is common in epidemiological studies (42, 43), which could affect risk estimates (especially if differential by consumption category or risk group). Furthermore, exposure and covariate data were collected solely at recruitment, so potential changes in the exposure or lifestyle variables occurring during the follow-up could not be taken into account. The fact that alcohol consumption was also evaluated retrospectively at participants' 20, 30, and 40 years of age let us integrate the past history and pattern of consumption in midlife, partially overcoming such limitation, while also allowing us to discriminate former drinkers from abstainers. Another potential limitation is the lack of information on apolipoprotein E (APOE) genotype since ε4 carriers have been shown to be more vulnerable to the deleterious effects of heavy alcohol consumption on cognition (44). Furthermore, we cannot rule out the possibility that occasional drinkers at baseline were misclassified as non-drinkers. However, this misclassification is likely minimal, and it is unlikely that these individuals were heavy alcohol consumers or that their inclusion meaningfully impacted our estimates. Also, some degree of underdiagnosis of dementia cases cannot be discarded, as case ascertainment relied on medical records, yet the universal coverage of the Spanish national health system might have reduced the impact of this potential limitation. The EPIC-Spain cohort was comprised of volunteers, not entirely representative of the general population, which limits the external validity of the results. Finally, participants did not undergo a baseline cognitive evaluation to discard pre-existing dementia or cognitive decline, which could lead to reverse causation. However, the review of clinical histories showed that less than 1% of the cases developed any form of dementia within the first 5 years of follow-up and excluding them had no measurable effect on the results.

Our results do not support that regular, low-dose alcohol consumption could have a neuroprotective effect against dementia or Alzheimer disease. While some evidence supports potential beneficial effects of moderate alcohol consumption, particularly wine (13, 15, 42, 45, 46), our study did not observe significant differences in dementia risk by type of alcoholic beverage consumed—wine, beer or liquors. Moreover, proposed biological pathways, such as the upregulation of heat shock proteins or cellular survival pathways (13), or potential benefits of social engagement (“social drinking”), remain speculative. Despite the biological plausibility of a “preconditioning” effect of alcohol at low doses, such hypotheses cannot be confirmed by observational designs alone.

On the other hand, heavy alcohol drinking was not found to exert a detrimental effect on dementia or dementia sub-type either, even when considering competing risks and lifetime consumption patterns. Ethanol is a neurotoxic, psychoactive and dependent-producing substance, further classified by the IARC as a Group 1 carcinogen, whereas several previous studies and meta-analyses have shown that heavy alcohol drinking led to worse cognitive results and higher dementia risk (12, 13, 47, 48). Chronic alcoholism is a main cause of the Wernicke-Korsakoff syndrome, a memory disturbance disorder secondary to thiamine (vitamin B_1_) depletion due to alcohol-induced malabsorption, and leads to lasting neurologic complications (49). In our study, however, a heavy alcohol consumption pattern remained not significantly associated with the risk dementia or Alzheimer disease even when accounting for non-dementia competing risks of death. Despite several other studies also reported a lack of association between heavy alcohol drinking and dementia risk (14), we can speculate that the most likely explanation for our null findings is the limited number of participants exhibiting excessive drinking habits in the cohort. The EPIC-Spain cohort comprised middle-age and elderly general population from a Mediterranean country, where alcohol was usually consumed with the meals, on a moderate and regular basis [the so-called “Mediterranean way of drinking” (50)] and where binge drinking patterns were infrequent. This limited proportion of participants with high heavy drinking habits, especially low among women—who represented two out of three cases of dementia–, could have reduced the power to detect a significant effect in these groups.

Notably, the protective association reported in the literature between moderate drinking and dementia appears to be attenuated in older adults. In a quantitative meta-analysis of prospective cohorts Xu et al. (15) found a higher protection for low-volume alcohol drinking in individuals under 60 years, while the effect was attenuated in those above 60. This age interaction may reflect both changes in alcohol metabolism with age and greater vulnerability to alcohol's neurotoxic effects in older adults. With aging, individuals typically exhibit reduced hepatic alcohol metabolism, lower body water content, and increased fat mass, all of which contribute to prolonged alcohol exposure and heightened central nervous system sensitivity, even at lower doses (51). Consequently, older adults may experience more adverse health effects from the same level of intake as younger individuals, counteracting the potential benefits of moderate drinking.

From a public health perspective, such age-related toxicity raises important concerns. Alcohol is the most common psychoactive substance consumed across the lifespan (52) and to a large extent, a culturally determined lifestyle (2, 45, 50), whereas it is a major cause of disease burden, estimated to account for 10% of global deaths (61.5% due to non-communicable diseases) and 3.7% of total DALYs (disability-adjusted life years) (53, 54). Thus, limiting excessive alcohol consumption has been recognized as an effective strategy to prevent the onset of chronic or neurodegenerative diseases in adult and older population. A previous study analyzing data from over 599,000 participants worldwide found the lowest mortality risk at approximately 100 g/week (21). Within the framework of the “Global Strategy to reduce the harmful use of alcohol,” the WHO has developed the “Global alcohol action plan 2022–2030” which aims at reducing harmful alcohol drinking, fostering the implementation of evidence-base interventions at global, national and community levels (55). In such a context, providing solid epidemiological evidence about whether moderate lifetime alcohol consumption could help to reduce the burden of chronic or neurodegenerative diseases remains a critical issue. Compared to previous studies, which often included fewer than 5,000 participants (13, 15), our analysis provides one of the most comprehensive evaluations of alcohol intake and dementia risk to date.

## Conclusion

Our results do not support a protective effect of moderate alcohol intake on dementia incidence, nor do they indicate a significant increase in risk from higher consumption in this Mediterranean midlife cohort. These results challenge previous evidence suggesting potential neuroprotective effects of alcohol consumption at low doses and support current WHO recommendations to limit alcohol intake as a prudent public health strategy given its related health burden. Future research should focus on life-course exposure trajectories, gene-environment interactions, and the integration of -omics data to better elucidate the role of alcohol in brain aging and dementia.

## Data Availability

The data will be made available from the authors upon reasonable request to the EPIC-Spain Steering Committee. Requests to access these datasets should be directed to Jose Maria Huerta, jmhuerta.carm@gmail.com.
