# Supplementary material for: Addressing common biases in the evaluation of lifetime alcohol consumption patterns and dementia risk: the EPIC-Spain dementia cohort
Source: Front Nutr. 2025 Oct 14;12:1671047. doi: 10.3389/fnut.2025.1671047 (PMC12560181; doi:10.3389/fnut.2025.1671047)
Supplement: Supplementary file 1 [file Data_Sheet_1.pdf]

frontiers

Supplementary Material

Addressing common biases in the evaluation of lifetime alcohol consumption patterns and dementia risk: the EPIC-Spain Dementia cohort

List of supplementary tables:

Supplementary table 1. Baseline characteristics of participants from the EPIC-Spain Dementia Cohort (N = 30,211) by baseline alcohol consumption categories.

Supplementary table 2. Baseline characteristics of participants from the EPIC-Spain Dementia Cohort (N = 30,211), by sex and dementia sub-type.

Supplementary table 3. Risk of overall dementia according to lifetime and baseline alcohol consumption categories in the EPIC-Spain Dementia Cohort, by smoking status.

Supplementary table 4. Risk of overall dementia according to lifetime and baseline alcohol consumption categories in the EPIC-Spain Dementia Cohort, by excess body weight.

Supplementary table 5. Sensitivity analyses of overall dementia risk according to lifetime and baseline alcohol consumption categories in the EPIC-Spain Dementia Cohort.

List of supplementary figures:

Figure S1. Risk of overall dementia according to baseline and mean lifetime alcohol consumption, by sex.

Figure S2. Risk of overall dementia according to baseline and mean lifetime alcohol consumption, by smoking status.

Figure S3. Risk of overall dementia according to mean baseline and lifetime alcohol consumption, by body mass index (BMI) categories.

Figure S4. Risk of dementia according to baseline and mean lifetime alcohol consumption, by dementia subtype.

Figure S5. Risk of overall dementia according to baseline and mean lifetime alcohol consumption, by type of alcoholic beverage.

Supplementary table 1. Baseline characteristics of participants from the EPIC-Spain Dementia Cohort (N = 30,211) by baseline alcohol consumption categories.

	Baseline alcohol consumption categories (g/d)													
	Never drinkers N = 6386		Former drinkers N = 4030		(0,6] (m) / (0,3] (w) N = 5612		(6,12] (m) / (3,12] (w) N = 4422		(12,24] N = 3736		(24,60] N = 4541		>60 N = 1484	
Baseline alcohol consumption (g/day), mean (s.d.)	0		0		1.6	(1.4)	7.2	(2.6)	17.1	(3.4)	38.3	(9.8)	85.1	(25.5)
Lifetime alcohol consumption (g/day), mean (s.d.)	0		8.8	(18.7)	6.8	(15.1)	11.3	(14.2)	23.9	(20.3)	47.6	(28.2)	88.2	(38.3)
Age at recruitment (y), mean (s.d.)	49.0	(8.4)	49.6	(8.1)	48.6	(8.0)	48.0	(8.0)	48.8	(7.7)	49.9	(7.3)	50.6	(6.8)
Women, n (%)	5989	(93.8)	2997	(74.4)	4086	(72.8)	3298	(74.6)	1754	(47.0)	694	(15.3)	24	(1.6)
Secondary education or higher, n (%)	1134	(17.8)	880	(21.8)	1439	(25.6)	1303	(29.5)	1197	(32.0)	1522	(33.5)	382	(25.7)
Body mass index (kg/m^2), mean (s.d.)	28.8	(4.9)	28.4	(4.5)	28.1	(4.3)	27.5	(4.2)	27.5	(3.8)	28.1	(3.4)	28.9	(3.4)
Waist circumference (cm), mean (s.d.)	88.9	(11.9)	91.0	(11.9)	90.7	(12.0)	89.5	(11.9)	92.7	(11.8)	97.6	(10.1)	101.1	(9.3)
Cigarette smoking, n (%)														
Never smoker	4898	(76.7)	2482	(61.6)	3559	(63.4)	2502	(56.6)	1687	(45.2)	1390	(30.6)	324	(21.8)
Former smoker	544	(8.5)	669	(16.6)	833	(14.8)	782	(17.7)	801	(21.4)	1178	(25.9)	341	(23.0)
Current smoker	942	(14.8)	878	(21.8)	1217	(21.7)	1136	(25.7)	1246	(33.4)	1970	(43.4)	819	(55.2)
Recreational physical activity (MET·h/week), mean (sd)	24.7	(20.3)	26.8	(22.2)	25.9	(22.6)	27.5	(22.4)	28.5	(24.3)	30.4	(25.6)	28.4	(25.2)
Household physical activity (MET·h/week), mean (sd)	101.9	(47.4)	80.8	(53.1)	79.2	(54.0)	76.7	(52.9)	54.4	(51.9)	28.6	(37.2)	18.1	(24.3)
Total energy intake (kcal/day), mean (s.d.)	1774	(565)	2007	(636)	2039	(625)	2111	(612)	2346	(660)	2691	(636)	3258	(730)
Energy from protein (%), mean (s.d.)	19.7	(3.5)	20.0	(3.3)	19.5	(2.9)	19.3	(2.8)	19.0	(2.5)	18.4	(2.2)	17.0	(2.0)
Energy from carbohydrates (%), mean (s.d.)	44.3	(6.5)	43.7	(6.6)	42.7	(6.1)	41.2	(5.9)	39.5	(5.9)	36.7	(5.9)	32.1	(5.5)
Energy from lipids (%), mean (s.d.)	36.0	(6.1)	36.3	(6.1)	37.2	(5.7)	36.9	(5.5)	35.9	(5.5)	34.4	(5.4)	32.0	(5.3)
Mediterranean diet score, mean (s.d.)[1]	8.3	(2.7)	8.3	(2.6)	8.2	(2.6)	8.2	(2.6)	8.1	(2.6)	8.0	(2.4)	7.1	(2.1)
Hypertension, n (%)	1298	(20.3)	935	(23.2)	1068	(19.0)	701	(15.9)	617	(16.5)	838	(18.5)	326	(22.0)
Hyperlipidemia, n (%)	949	(14.9)	784	(19.5)	922	(16.4)	744	(16.8)	706	(18.9)	1092	(24.1)	400	(27.0)
Post-menopausal, n (%)[2]	2409	(40.2)	1218	(40.6)	1451	(35.5)	1045	(31.7)	506	(28.9)	195	(28.1)	6	(25.0)
Oral contraceptive use (ever), n (%)[2]	2305	(38.5)	1197	(39.9)	1813	(44.4)	1537	(46.6)	812	(46.3)	357	(51.4)	9	(37.5)
Hormonal replacement therapy use (ever), n (%)[2]	550	(9.2)	294	(9.8)	366	(9.0)	287	(8.7)	152	(8.7)	64	(9.2)	2	(8.3)

[1] Adapted relative Mediterranean diet score (arMED).

[2] Women only.

Supplementary table 2. Baseline characteristics of participants from the EPIC-Spain Dementia Cohort (N = 30,211), by sex and dementia sub-type.

	All						Men						Women					
	Non dementia N = 29097		Alzheimer N= 774		Non-Alzheimer N= 340		Non dementia N = 10998		Alzheimer N = 235		Non-Alzheimer N = 136		Non dementia N = 18099		Alzheimer N = 539		Non-Alzheimer N = 204	
Baseline alcohol consumption (g/day), mean (s.d.)	13.5	(22.0)	10.1	(20.0)	13.9	(32.1)	28.6	(28.4)	27.2	(28.7)	30.7	(45.4)	4.3	(8.2)	2.7	(6.1)	2.6	(5.9)
Lifetime alcohol consumption (g/day), mean (s.d.)	18.6	(29.2)	14.7	(27.1)	19.5	(30.7)	41.9	(36.0)	41.4	(36.4)	42.7	(37.2)	4.5	(6.9)	3.1	(5.2)	3.9	(6.8)
Age at recruitment (y), mean (s.d.)	48.7	(7.8)	58.0	(5.6)	57.9	(6.0)	50.2	(7.1)	58.1	(5.2)	57.7	(5.9)	47.8	(8.1)	58.0	(5.7)	58.0	(6.0)
Women, n (%)	18099	(62.2)	539	(69.6)	204	(60.0)	0	-	0	-	0	-	100	-	100	-	100	-
Secondary education or higher, n (%)	7739	(26.6)	83	(10.7)	35	(10.3)	3973	(36.1)	45	(19.2)	28	(20.6)	3766	(20.8)	38	(7.1)	7	(3.4)
Body mass index (kg/m^2), mean (s.d.)	28.1	(4.3)	29.6	(4.1)	29.7	(4.3)	28.4	(3.4)	29.0	(3.5)	28.9	(3.5)	28.0	(4.7)	29.9	(4.3)	30.2	(4.7)
Waist circumference (cm), mean (s.d.)	91.9	(12.1)	94.8	(10.3)	96.7	(10.8)	99.6	(9.1)	101.1	(8.7)	101.6	(9.0)	87.2	(11.3)	92.1	(9.8)	93.5	(10.7)
Cigarette smoking, n (%)																		
Never smoker	16037	(55.1)	583	(75.3)	222	(65.3)	3266	(29.7)	87	(37.0)	42	(30.9)	12771	(70.6)	496	(92.0)	180	(88.2)
Former smoker	5011	(17.2)	84	(10.9)	53	(15.6)	3170	(28.8)	71	(30.2)	45	(33.1)	1841	(10.2)	13	(2.4)	8	(3.9)
Current smoker	8037	(27.6)	107	(13.8)	64	(18.8)	4558	(41.4)	77	(32.8)	49	(36.0)	3479	(19.2)	30	(5.6)	15	(7.4)
Recreational physical activity (MET·h/week), mean (sd)	27.1	(22.9)	26.0	(23.2)	28.0	(23.9)	31.4	(26.7)	33.8	(28.9)	35.0	(28.4)	24.5	(19.9)	22.6	(19.4)	23.3	(19.0)
Household physical activity (MET·h/week), mean (sd)	69.8	(55.1)	82.3	(55.8)	71.8	(55.1)	17.8	(21.7)	19.0	(22.5)	20.6	(23.7)	101.5	(44.1)	109.9	(41.8)	106.0	(41.9)
Total energy intake (kcal/day), mean (s.d.)	2191	(729)	2002	(704)	2062	(746)	2652	(714)	2569	(680)	2489	(718)	1911	(581)	1755	(556)	1778	(619)
Energy from protein (%), mean (s.d.)	19.2	(3.0)	19.3	(3.1)	19.1	(2.9)	18.8	(2.6)	18.6	(2.6)	18.5	(2.5)	19.5	(3.2)	19.6	(3.3)	19.4	(3.1)
Energy from carbohydrates (%), mean (s.d.)	41.0	(6.9)	43.1	(7.3)	42.7	(7.9)	38.9	(7.0)	39.5	(7.8)	40.2	(8.0)	42.4	(6.6)	44.6	(6.4)	44.4	(7.4)
Energy from lipids (%), mean (s.d.)	36.0	(5.9)	34.7	(6.0)	34.4	(6.5)	35.0	(5.7)	34.9	(5.8)	33.2	(6.0)	36.6	(5.9)	34.7	(6.0)	35.2	(6.6)
Mediterranean diet score, mean (s.d.)[1]	8.1	(2.6)	8.4	(2.5)	8.1	(2.6)	8.2	(2.5)	8.4	(2.4)	8.4	(2.5)	8.1	(2.6)	8.4	(2.5)	8.0	(2.6)
Hypertension, n (%)	5452	(18.7)	225	(29.1)	106	(31.2)	2221	(20.2)	62	(26.4)	33	(24.3)	3231	(17.9)	163	(30.2)	73	(35.8)
Hyperlipidemia, n (%)	5308	(18.2)	204	(26.4)	85	(25.0)	2662	(24.2)	54	(23.0)	29	(21.3)	2646	(14.6)	150	(27.8)	56	(27.5)
Post-menopausal, n (%)[2]	-		-		-		-		-		-		6215	(34.3)	449	(83.3)	166	(81.4)
Oral contraceptive use (ever), n (%)[2]	-		-		-		-		-		-		7889	(43.6)	110	(20.4)	31	(15.2)
Hormonal replacement therapy use (ever), n (%)[2]	-		-		-		-		-		-		1616	(8.9)	68	(12.6)	31	(15.2)

[1] Adapted relative Mediterranean diet score (arMED).

[2] Women only.

Supplementary table 3. Risk of overall dementia according to lifetime and baseline alcohol consumption categories in the EPIC-Spain Dementia Cohort, by smoking status.

	Never smokers				Ever smokers			
	Person-years	Cases	SHR	95% CI	Person-years	Cases	SHR	95% CI
Average baseline alcohol consumption (g/d)								
Never drinkers	115611	292	1 (ref.)		35088	19	1 (ref.)	
Former drinkers	56869	118	0.89	(0.71, 1.12)	34877	46	1.41	(0.81, 2.47)
(0,6] (m) / (0,3] (w)	82200	162	0.96	(0.79, 1.18)	46877	40	1.14	(0.65, 2.02)
(6,12] (m) / (3,12] (w)	57055	109	1.03	(0.81, 1.29)	43951	39	1.24	(0.71, 2.15)
(12,24]	38425	61	0.89	(0.66, 1.19)	46119	46	1.09	(0.62, 1.90)
(24,60]	31253	43	0.74	(0.51, 1.07)	69613	91	1.19	(0.69, 2.05)
(60,96] (m) / >60 (w)	6031	17	1.34	(0.78, 2.30)	18812	17	0.75	(0.37, 1.52)
>96 (m)	1173	3	1.04	(0.33, 3.30)	6087	10	1.24	(0.55, 2.80)
Current versus never	331747	687	0.95	(0.80, 1.14)	266547	262	1.13	(0.68, 1.88)
Average lifetime alcohol consumption (g/d)								
Never drinkers	115611	292	1 (ref.)		35088	19	1 (ref.)	
Former drinkers	56869	118	0.90	(0.72, 1.13)	34877	46	1.40	(0.79, 2.45)
(0,6] (m) / (0,3] (w)	70701	132	0.92	(0.75, 1.14)	33313	26	1.30	(0.71, 2.38)
(6,12] (m) / (3,12] (w)	63469	105	0.91	(0.71, 1.15)	41778	27	1.18	(0.66, 2.11)
(12,24]	37629	88	1.25	(0.97, 1.63)	41214	40	1.12	(0.64, 1.98)
(24,60]	31180	42	0.78	(0.52, 1.17)	70461	87	1.07	(0.61, 1.87)
(60,96] (m) / >60 (w)	9796	21	0.91	(0.55, 1.53)	29930	39	0.97	(0.52, 1.80)
>96 (m)	3360	7	0.82	(0.36, 1.86)	14764	24	1.20	(0.61, 2.37)
Ever versus never	388615	805	0.94	(0.80, 1.11)	301424	308	1.21	(0.74, 2.00)
Lifetime pattern of alcohol consumption								
Never drinkers	115611	292	1 (ref.)		35088	19	1 (ref.)	
Former light drinkers	54858	114	0.89	(0.71, 1.12)	29636	38	1.51	(0.85, 2.67)
Former heavy drinkers	2010	4	0.93	(0.35, 2.52)	5241	8	0.98	(0.42, 2.32)
Light drinkers	49991	102	0.98	(0.78, 1.23)	19373	17	1.66	(0.85, 3.23)
Never heavy drinkers	135011	236	0.96	(0.79, 1.16)	130092	120	1.08	(0.65, 1.79)
Periodically heavy drinkers	26492	46	0.87	(0.61, 1.26)	66729	92	1.10	(0.64, 1.90)
Always heavy drinkers	4641	11	1.11	(0.58, 2.15)	15265	14	0.72	(0.34, 1.52)

SHR: Sub-hazard ratio. CI: confidence Interval.
Models adjusted by center, sex, educational level, energy intake (alcohol excluded), BMI categories, sex x BMI interaction, elevated waist circumference, household and recreational physical activity, hypertension, hyperlipidemia, and Mediterranean diet score (arMED).

Supplementary table 4. Risk of dementia according to lifetime and baseline alcohol consumption categories in the EPIC-Spain Dementia Cohort, by excess body weight.

	Normal weight				Overweight or obese			
	Person-years	Cases	SHR	95% CI	Person-years	Cases	SHR	95% CI
Average baseline alcohol consumption (g/d)								
Never drinkers	34590	25	1 (ref.)		116151	287	1 (ref.)	
Former drinkers	21078	26	1.47	(0.84, 2.57)	70691	138	0.90	(0.73, 1.11)
(0,6] (m) / (0,3] (w)	32319	34	1.66	(0.96, 2.87)	96827	168	0.89	(0.73, 1.09)
(6,12] (m) / (3,12] (w)	29746	15	0.87	(0.45, 1.68)	71308	133	1.07	(0.86, 1.33)
(12-24]	22422	12	0.75	(0.38, 1.50)	62171	95	0.93	(0.72, 1.20)
(24-60]	16974	16	0.82	(0.40, 1.66)	83965	118	0.90	(0.69, 1.18)
(60,96] (m) / >60 (w)	2770	2	0.44	(0.09, 2.11)	22073	32	0.90	(0.60, 1.36)
>96 (m)	596	1	0.60	(0.08, 4.53)	6664	12	1.08	(0.59, 1.99)
Current versus never	139417	105	1.19	(0.72, 1.96)	459159	845	0.95	(0.80, 1.12)
Average lifetime alcohol consumption (g/d)								
Never drinkers	34590	25	1 (ref.)		116151	287	1 (ref.)	
Former drinkers	21078	26	1.50	(0.85, 2.63)	70691	138	0.90	(0.73, 1.11)
(0,6] (m) / (0,3] (w)	28429	19	1.18	(0.63, 2.21)	75678	139	0.92	(0.75, 1.13)
(6,12] (m) / (3,12] (w)	33849	19	1.15	(0.62, 2.14)	71473	113	0.90	(0.72, 1.14)
(12,24]	21211	18	1.14	(0.61, 2.13)	57657	110	1.15	(0.90, 1.48)
(24,60]	15380	14	0.79	(0.37, 1.72)	86309	115	0.88	(0.66, 1.17)
(60,96] (m) / >60 (w)	4187	8	0.98	(0.38, 2.53)	35539	92	0.84	(0.58, 1.20)
>96 (m)	1772	2	0.68	(0.14, 3.31)	16352	29	1.00	(0.64, 1.56)
Ever versus never	160495	131	1.22	(0.76, 1.94)	529850	983	0.93	(0.80, 1.10)
Lifetime pattern of alcohol consumption								
Never drinkers	34590	25	1 (ref.)		116151	287	1 (ref.)	
Former light drinkers	19639	25	1.65	(0.95, 2.88)	64879	127	0.89	(0.71, 1.10)
Former heavy drinkers	1440	1	0.55	(0.08, 3.87)	5811	11	0.87	(0.46, 1.64)
Light drinkers	18485	11	1.04	(0.49, 2.20)	50924	108	1.02	(0.81, 1.27)
Never heavy drinkers	70585	51	1.13	(0.67, 1.88)	194688	305	0.92	(0.76, 1.11)
Periodically heavy drinkers	13386	15	1.00	(0.47, 2.11)	79860	123	0.90	(0.69, 1.18)
Always heavy drinkers	2370	3	0.88	(0.23, 3.28)	17536	22	0.75	(0.46, 1.22)

Normal weight: body mass index (BMI) < 25 kg/m^2. Overerweight or obese: BMI ≥ 25 kg/m^2.

SHR: Sub-hazard ratio. CI: confidence Interval.

Models adjusted by center, sex, educational level, energy intake (alcohol excluded), smoking, elevated waist circumference, household and recreational physical activity, hypertension, hyperlipidemia, and Mediterranean diet score (arMED).

Supplementary table 5. Sensitivity analyses of overall dementia risk according to lifetime and baseline alcohol consumption categories in the EPIC-Spain Dementia Cohort.

	Plausible energy reporters (N = 21,021)				45 ≤ Age < 65 (N = 19,274)				High confidence of dementia diagnosis (N= 30,008)				All three criteria (N = 13,373)			
	Person-years	Cases	SHR	95% CI	Person-years	Cases	SHR	95% CI	Person-years	Cases	SHR	95% CI	Person-years	Cases	SHR	95% CI
Average baseline alcohol consumption (g/d)																
Never drinkers	105893	203	1 (ref.)		92836	287	1 (ref.)		149649	251	1 (ref.)		65156	155	1 (ref.)	
Former drinkers	64713	113	0.97	(0.77, 1.24)	59468	150	0.93	(0.76, 1.15)	91200	131	0.91	(0.73, 1.13)	42135	84	0.91	(0.69, 1.20)
(0,6] (m) / (0,3] (w)	93929	146	1.01	(0.81, 1.26)	78334	186	0.95	(0.79, 1.16)	128599	171	0.97	(0.79, 1.19)	56875	112	0.96	(0.75, 1.23)
(6,12] (m) / (3,12] (w)	72647	101	1.00	(0.78, 1.28)	58072	135	1.00	(0.80, 1.24)	100487	117	0.96	(0.77, 1.21)	41623	75	0.89	(0.67, 1.19)
(12,24]	60574	78	0.91	(0.68, 1.20)	52856	100	0.87	(0.68, 1.11)	84327	91	0.87	(0.67, 1.14)	38372	62	0.84	(0.61, 1.16)
(24,60]	68945	95	0.93	(0.69, 1.26)	68952	125	0.84	(0.65, 1.09)	100481	109	0.82	(0.62, 1.08)	47494	74	0.84	(0.60, 1.18)
(60,96] (m) / >60 (w)	13170	18	0.89	(0.52, 1.51)	17968	34	0.84	(0.57, 1.26)	24749	28	0.77	(0.50, 1.19)	9358	14	0.80	(0.44, 1.44)
>96 (m)	1817	3	0.84	(0.27, 2.61)	5301	12	0.95	(0.51, 1.75)	7260	13	1.11	(0.61, 2.01)	1379	3	0.97	(0.31, 3.02)
Current versus never	416976	644	1.00	(0.82, 1.21)	374320	879	0.95	(0.80, 1.13)	595552	780	0.95	(0.79, 1.13)	260258	495	0.92	(0.74, 1.14)
Average lifetime alcohol consumption (g/d)																
Never drinkers	105893	203	1 (ref.)		92836	287	1 (ref.)		149649	251	1 (ref.)		65156	155	1 (ref.)	
Former drinkers	64713	113	0.99	(0.78, 1.25)	59468	150	0.94	(0.76, 1.16)	91200	131	0.91	(0.73, 1.13)	42135	84	0.92	(0.70, 1.21)
(0,6] (m) / (0,3] (w)	76528	112	0.97	(0.77, 1.23)	60265	139	0.91	(0.74, 1.12)	103613	130	0.95	(0.76, 1.18)	43992	81	0.90	(0.69, 1.18)
(6,12] (m) / (3,12] (w)	73713	90	0.92	(0.71, 1.19)	58518	125	0.93	(0.75, 1.17)	104904	110	0.89	(0.71, 1.13)	41250	71	0.88	(0.66, 1.17)
(12,24]	54760	87	1.12	(0.85, 1.48)	48932	117	1.10	(0.87, 1.40)	78493	106	1.08	(0.84, 1.39)	34413	66	1.00	(0.73, 1.37)
(24,60]	72037	94	0.96	(0.69, 1.33)	69834	122	0.83	(0.63, 1.10)	101316	108	0.80	(0.60, 1.09)	49604	75	0.86	(0.59, 1.26)
(60,96] (m) / >60 (w)	24829	40	0.97	(0.64, 1.47)	30403	58	0.83	(0.59, 1.18)	39573	51	0.78	(0.54, 1.13)	18938	33	0.87	(0.55, 1.38)
>96 (m)	9216	18	1.22	(0.71, 2.09)	13532	31	0.99	(0.64, 1.53)	18004	24	0.80	(0.50, 1.29)	6904	14	1.04	(0.57, 1.91)
Ever versus never	481689	757	0.98	(0.82, 1.17)	433788	1029	0.94	(0.80, 1.10)	686752	911	0.93	(0.79, 1.10)	302392	579	0.91	(0.74, 1.12)
Lifetime pattern of alcohol consumption																
Never drinkers	105893	203	1 (ref.)		92836	287	1 (ref.)		149649	251	1 (ref.)		65156	155	1 (ref.)	
Former light drinkers	59802	106	1.00	(0.78, 1.27)	53913	138	0.94	(0.76, 1.16)	84032	123	0.92	(0.74, 1.16)	38491	80	0.94	(0.72, 1.24)
Former heavy drinkers	4910	7	0.69	(0.32, 1.51)	5556	12	0.90	(0.49, 1.63)	7168	8	0.65	(0.31, 1.35)	3643	4	0.52	(0.19, 1.43)
Light drinkers	50992	86	1.07	(0.83, 1.37)	40549	106	1.00	(0.80, 1.25)	69056	98	1.03	(0.81, 1.30)	29810	62	0.99	(0.74, 1.33)
Never heavy drinkers	189307	251	0.93	(0.76, 1.15)	159358	331	0.92	(0.77, 1.11)	264133	294	0.90	(0.74, 1.09)	113968	192	0.86	(0.68, 1.09)
Periodically heavy drinkers	62014	92	0.94	(0.69, 1.28)	67289	130	0.88	(0.68, 1.15)	92864	116	0.85	(0.64, 1.12)	45146	77	0.88	(0.62, 1.24)
Always heavy drinkers	8770	12	0.87	(0.46, 1.64)	14288	25	0.78	(0.49, 1.23)	19850	21	0.69	(0.42, 1.14)	6178	9	0.74	(0.36, 1.54)

SHR: Sub-hazard ratio. CI: confidence Interval.

Models adjusted by center, sex, educational level, energy intake (alcohol excluded), smoking, BMI categories, sex x BMI interaction, elevated waist circumference, household and recreational physical activity, hypertension, hyperlipidemia, and Mediterranean diet score (arMED).

Supplementary figures.

Figure S1. Risk of overall dementia according to baseline and mean lifetime alcohol consumption, by sex.

Dementia risks were estimated by means of sub-hazard ratios derived from Fine & Gray competing risk models with age as the timescale and non-dementia deaths as competing events. Alcohol intake variables were transformed using restricted cubic splines with 3 degrees of freedom and equally spaced knots. Models were adjusted by center, educational level, energy from non-alcoholic sources, body mass index category (normal weight, overweight, obese), elevated waist circumference (>102 cm (men)/>88 cm (women)), household and recreational physical activity (METh/week), self-reported hypertension or hyperlipidemia, and adapted relative Mediterranean Diet score (arMED). Lifetime models were further adjusted by duration of alcohol consumption and time since quitting alcohol (years). Reference was set at 0 g/d of alcohol consumption. Former drinkers were excluded.

Figure S2. Risk of overall dementia according to baseline and mean lifetime alcohol consumption, by smoking status.

Dementia risks were estimated by means of sub-hazard ratios derived from Fine & Gray competing risk models with age as the timescale and non-dementia deaths as competing events. Alcohol intake variables were transformed using restricted cubic splines with 3 degrees of freedom and equally spaced knots. Models were adjusted by center, sex, educational level, energy from non-alcoholic sources, body mass index category (normal weight, overweight, obese), sex *x* body mass index interaction, elevated waist circumference (>102 cm (men)/>88 cm (women)), household and recreational physical activity (METh/week), self-reported hypertension or hyperlipidemia, and adapted relative Mediterranean Diet score (arMED). Lifetime models were further adjusted by duration of alcohol consumption and time since quitting alcohol (years). Reference was set at 0 g/d of alcohol consumption. Former drinkers were excluded.

Figure S3. Risk of overall dementia according to mean baseline and lifetime alcohol consumption, by body mass index (BMI) categories.

Dementia risks were estimated by means of sub-hazard ratios derived from Fine & Gray competing risk models with age as the timescale and non-dementia deaths as competing events. Normal weight was defined as BMI<25 kg/m^2, and excess body weight was defined as BMI≥25 kg/m^2. Alcohol intake variables were transformed using restricted cubic splines with 3 degrees of freedom and equally spaced knots. Models were adjusted by center, sex, educational level, energy from non-alcoholic sources, smoking status (never, former, current), elevated waist circumference (>102 cm (men)/>88 cm (women)), household and recreational physical activity (METh/week), self-reported hypertension or hyperlipidemia, and adapted relative Mediterranean Diet score (arMED). Lifetime models were further adjusted by duration of alcohol consumption and time since quitting alcohol (years). Reference was set at 0 g/d of alcohol consumption. Former drinkers were excluded.

Figure S4. Risk of dementia according to baseline and mean lifetime alcohol consumption, by dementia subtype.

Risks of dementia sub-types (Alzheimer and non-Alzheimer) were estimated by means of sub-hazard ratios derived from Fine & Gray competing risk models with age as the timescale and non-dementia deaths as competing events. Alcohol intake variables were transformed using restricted cubic splines with 3 degrees of freedom and equally spaced knots. Models were adjusted by center, sex, educational level, energy from non-alcoholic sources, smoking status (never, former, current), body mass index category (normal weight, overweight, obese), sex *x* body mass index interaction, elevated waist circumference (>102 cm (men)/>88 cm (women)), household and recreational physical activity (METh/week), self-reported hypertension or hyperlipidemia, and adapted relative Mediterranean Diet score (arMED). Lifetime models were further adjusted by duration of alcohol consumption and time since quitting alcohol (years). Reference was set at 0 g/d of alcohol consumption. Former drinkers were excluded.

Figure S5. Risk of overall dementia according to baseline and mean lifetime alcohol consumption, by type of alcoholic beverage.

Dementia risks were estimated by means of sub-hazard ratios derived from Fine & Gray competing risk models with age as the timescale and non-dementia deaths as competing events. Alcohol intake variables were transformed using restricted cubic splines with 3 degrees of freedom and equally spaced knots. Models were built separately for each type of beverage and adjusted for energy from other sources of alcohol and macronutrients, center, sex, educational level, energy from non-alcoholic sources, smoking status (never, former, current), body mass index category (normal weight, overweight, obese), sex *x* body mass index interaction, elevated waist circumference (>102 cm (men)/>88 cm (women)), household and recreational physical activity (METh/week), self-reported hypertension or hyperlipidemia, and adapted relative Mediterranean Diet score (arMED). Lifetime models were further adjusted by duration of alcohol consumption and time since quitting alcohol (years). Reference was set at 0 g/d of alcohol consumption. Former drinkers were excluded.

Figure S1

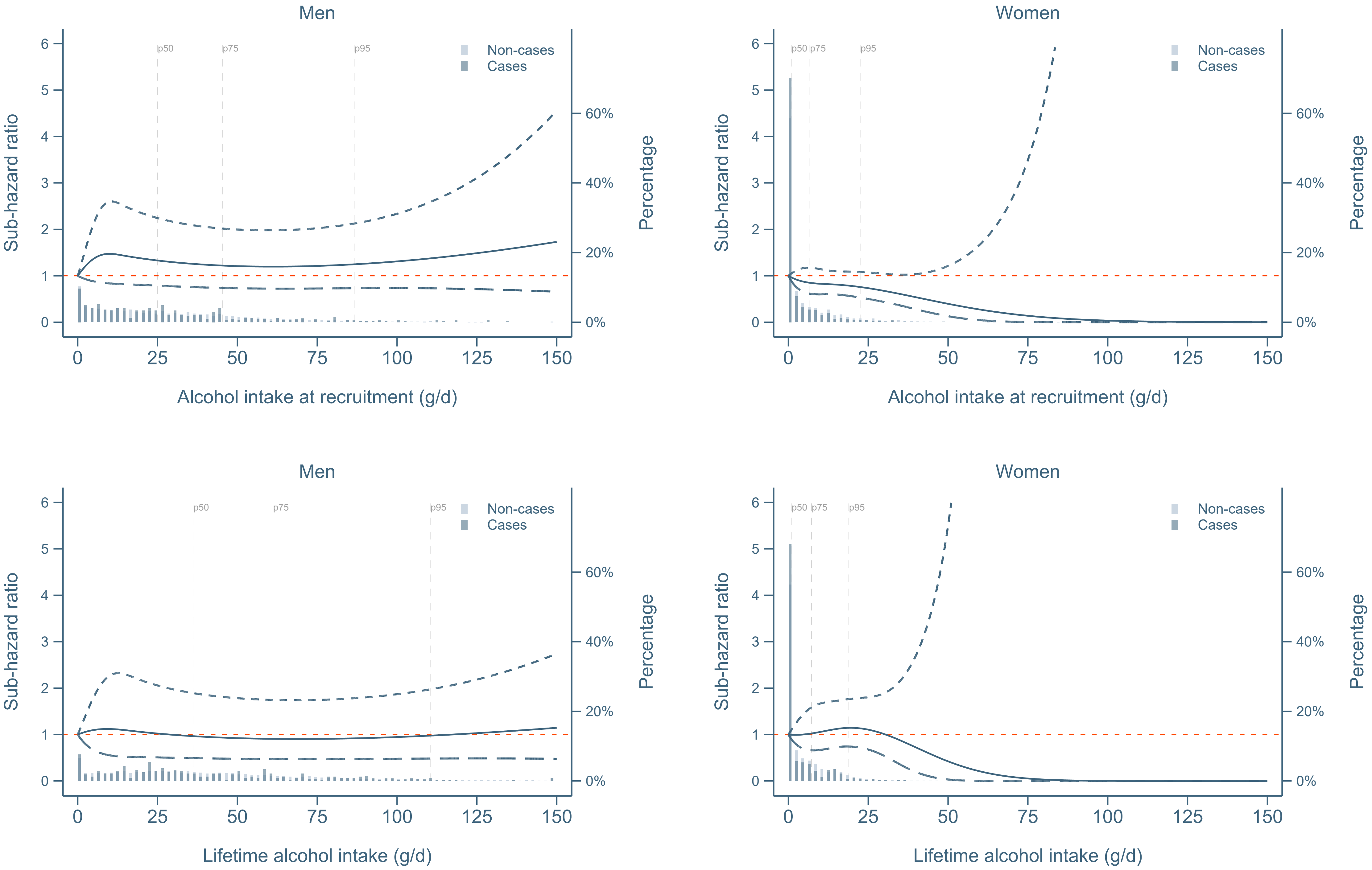

Men
Women
Men
Women
Sub-hazard ratio
Percentage
Non-cases
Cases
p50
p75
p95
0
1
2
3
4
5
6
0%
20%
40%
60%
0
25
50
75
100
125
150
Alcohol intake at recruitment (g/d)
Lifetime alcohol intake (g/d)

Figure S2

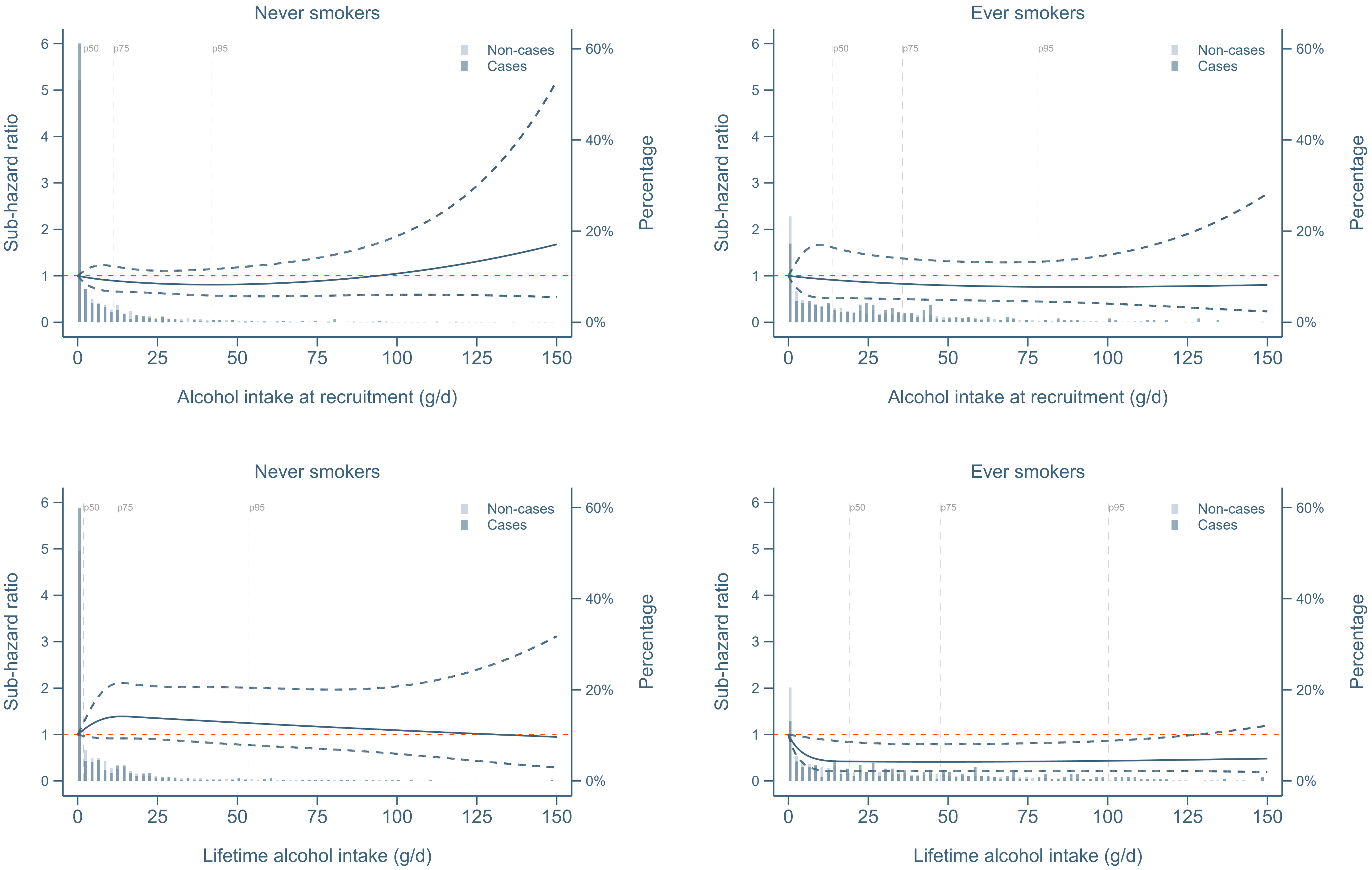
Never smokers
Ever smokers
Never smokers
Ever smokers
Sub-hazard ratio
Percentage
Non-cases
Cases
p50
p75
p95
0
1
2
3
4
5
6
0%
20%
40%
60%
0
25
50
75
100
125
150
Alcohol intake at recruitment (g/d)
Lifetime alcohol intake (g/d)

Figure S3

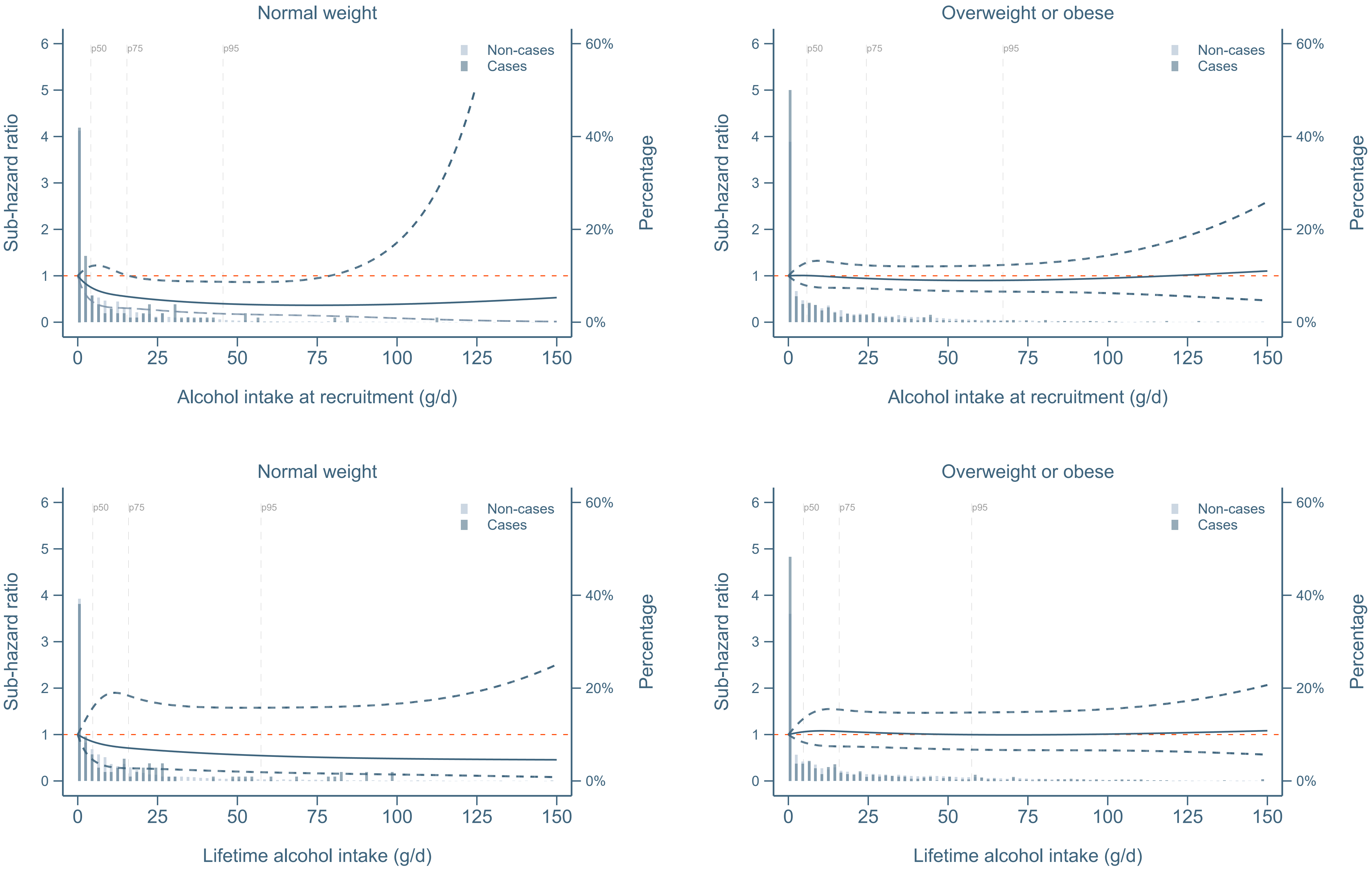

Normal weight
Overweight or obese
Non-cases
Cases
p50
p75
p95
Sub-hazard ratio
Percentage
0
1
2
3
4
5
6
0%
20%
40%
60%
0
25
50
75
100
125
150
Alcohol intake at recruitment (g/d)
Normal weight
Overweight or obese
Lifetime alcohol intake (g/d)

Figure S4

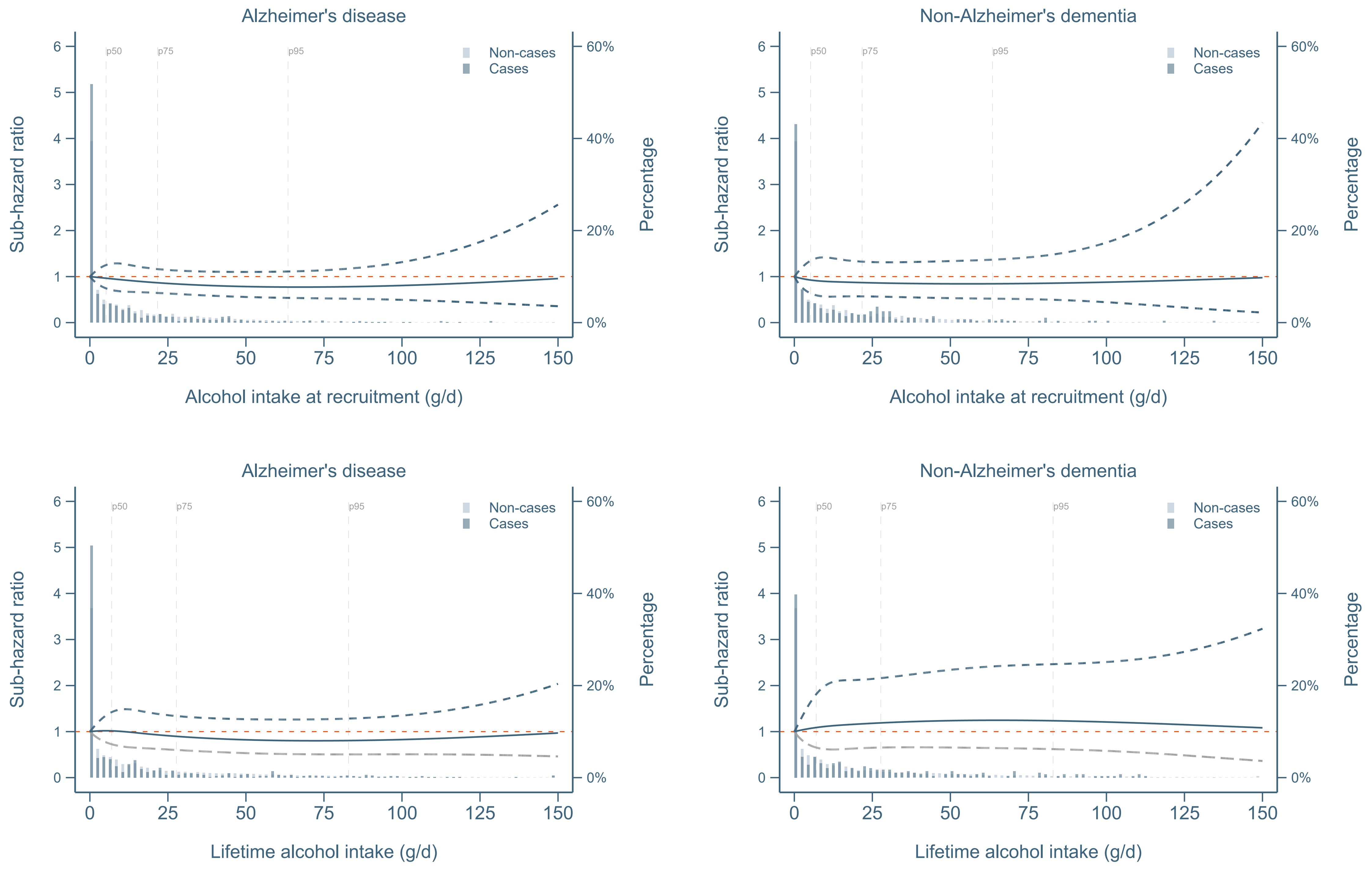
Alzheimer's disease
Non-Alzheimer's dementia
Alzheimer's disease
Non-Alzheimer's dementia
Sub-hazard ratio
Percentage
Non-cases
Cases
p50
p75
p95
0
1
2
3
4
5
6
0%
20%
40%
60%
0
25
50
75
100
125
150
Alcohol intake at recruitment (g/d)
Lifetime alcohol intake (g/d)

Figure S5

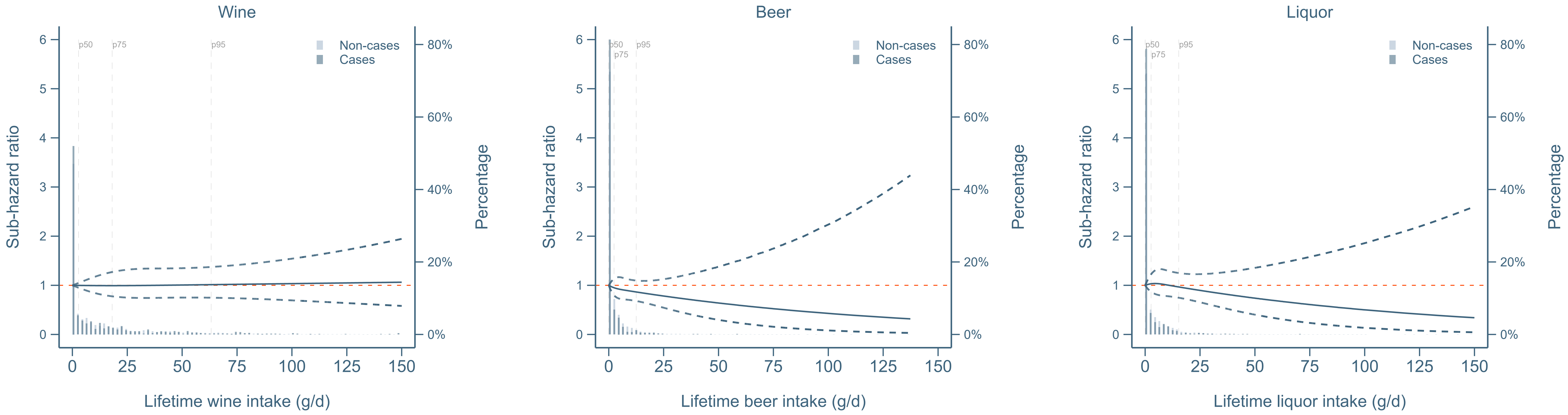
Wine
Beer
Liquor
Sub-hazard ratio
Percentage
Non-cases
Cases
p50
p75
p95
0
1
2
3
4
5
6
0%
20%
40%
60%
80%
0
25
50
75
100
125
150
Lifetime wine intake (g/d)
Lifetime beer intake (g/d)
Lifetime liquor intake (g/d)
